# The effect of environmental stressors on growth in fish and its endocrine control

**DOI:** 10.3389/fendo.2023.1109461

**Published:** 2023-03-30

**Authors:** Luis Fabián Canosa, Juan Ignacio Bertucci

**Affiliations:** ^1^ Instituto Tecnológico Chascomús (INTECH), CONICET-EByNT-UNSAM, Chascomús, Argentina; ^2^ Centro Oceanográfico de Vigo, Instituto Español de Oceanografía - Consejo Superior de Investigaciones Científicas (IEO-CSIC), Vigo, Spain

**Keywords:** climate change, environmental pollutants, growth hormone, IGF, somatic growth, feeding, teleost

## Abstract

Fish body growth is a trait of major importance for individual survival and reproduction. It has implications in population, ecology, and evolution. Somatic growth is controlled by the GH/IGF endocrine axis and is influenced by nutrition, feeding, and reproductive-regulating hormones as well as abiotic factors such as temperature, oxygen levels, and salinity. Global climate change and anthropogenic pollutants will modify environmental conditions affecting directly or indirectly fish growth performance. In the present review, we offer an overview of somatic growth and its interplay with the feeding regulatory axis and summarize the effects of global warming and the main anthropogenic pollutants on these endocrine axes.

## 1 Introduction

Fish growth rate is an important trait with ecological, evolutionary, and conservation implications (1, 2). Anthropogenic stressors such as global climate change, which imply temperature rise, salinity change, and ocean acidification; ecosystem disturbance; and selective pressure from capture have shown a rapid effect on the growth rate of several fish species (2–10). During the last century, the mean global temperatures have risen approximately 1.1°C concurrently with increasing anthropogenic greenhouse gas emissions (11). Temperature is one of the main abiotic factors affecting body size in fish (12–14). It has been described that increasing temperature induces a reduction in body size of ectotherm organisms, such as fish (8, 15–18). Beyond the passionate discussion about the underlying mechanism to explain this fact (19, 20), it is forecasted that body size will be reduced in any scenario of global warming. Reduction of body size might have important deleterious effects not only in the reduction of species fitness but also perturbation of energy and nutrient web, ecosystem, and economic losses (1, 2, 7, 21, 22). In this regard, it would be necessary to understand the effects of anthropogenic stressors on somatic growth endocrine regulation.

**Figure 1:**
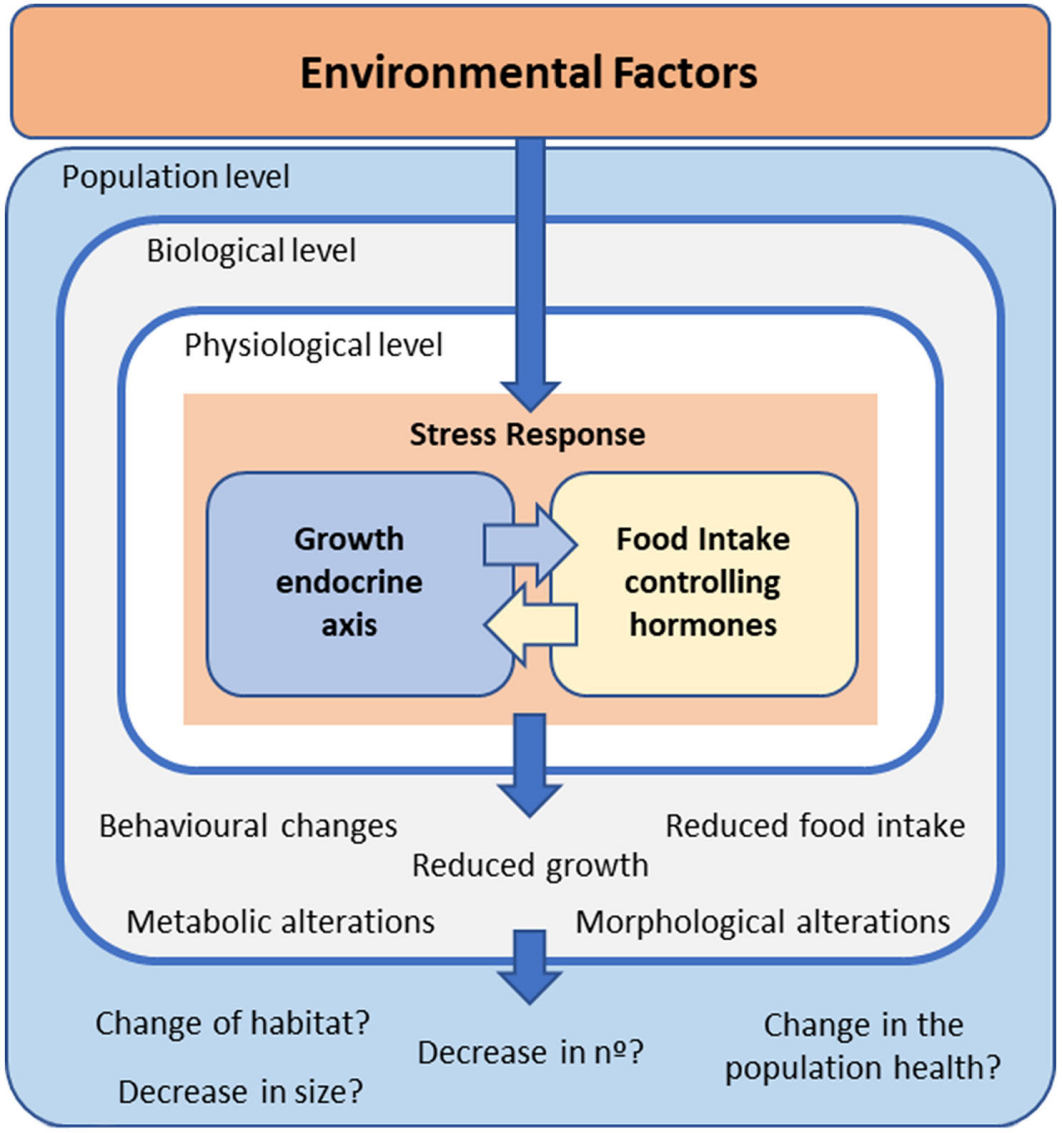
Schematic representation of how environmental factors activate a stress response in fish, which includes modifications in the interrelated growth – and food intake –endocrine axes. The possible consequences at different levels of biological organization are also mentioned.

Body growth is promoted by growth hormone–insulin‐like growth factors, the so-called GH/IGF axis or somatic growth axis (23–27). This endocrine axis is integrated and influenced by reproduction, feeding, and energy balance (23, 25, 28–30). Therefore, fish growth is affected by a wide range of biotic and abiotic factors, such as temperature (12, 13), nutritional state and energy metabolism (30–32), intra- and interspecies interactions (33, 34), and sex and reproduction (35–37). Thus, pollutants could interfere with this hormonal assembly that impacts the growth rate and performance of fish, potentially affecting their populations and the entire ecosystem (**Figure 1**).

The main goal of this review is to provide an overview and update on the effects of several abiotic anthropogenic factors on GH and food intake regulation. By summarizing the current knowledge on this topic, this review aims to serve as a resource for those involved in research related to understanding the effects of global warming and pollutants on individuals and population growth and the energy web.

## 2 Interrelationship between growth and food intake in fish

The endocrine and neuroendocrine regulation of somatic growth in teleost fish has been extensively reviewed (23, 24, 26–29, 38–40). The main factors regulating growth are growth hormone (GH), the insulin-like growth factors (IGFs), and their respective receptors (GHRs and IGFRs). Systemic IGFs, derived mainly from liver sources, in turn, act in a negative feedback fashion to suppress GH. GH belongs to the GH/prolactin (PRL)/somatolactin (SL) family (23, 39, 41). GH, produced in and released by the pituitary gland, is largely responsible for endocrine growth promotion during postnatal growth. Once in the circulation, according to the classical somatomedin hypothesis (42–44), the main endocrine action of GH is stimulating hepatic IGF production, which is the primary inducer of cell proliferation and differentiation (45, 46). IGFs act on target tissues in either an autocrine, paracrine, or endocrine manner (43, 47–49) stimulating many biological processes, including protein synthesis and turnover and cell proliferation and differentiation, among others (44, 50).

Furthermore, GH acts directly on different target tissues including the muscle and adipose tissue, to regulate physiological processes associated with protein synthesis and metabolism (40). GH also induces the local production of IGFs to regulate, in a paracrine way, cell proliferation and tissue growth, as shown in mammals (51). In addition to growth promotion, GH is a pleiotropic hormone also involved in several important processes such as nutrition, metabolism, reproduction, neuroprotection, immunity, osmoregulation, and social behavior (23, 28, 39, 40, 45, 49, 52–55). Consequently, the neuroendocrine regulation of GH is influenced by several peptides and neurotransmitters also involved in the regulation of either food intake or reproduction [for review, see (23, 28, 29, 53)], suggesting a close integration of different endocrine axes (**Table 1**).

**Table 1:** Summary of interacting factors linking growth and food intake in fish.

Factor	Endocrine axis	Main function	Interaction with other systems	Ref.
GHRH	Growth	Stimulation of GH release	Stimulation of food intake	(23, 28, 29)
PACAP	Growth	Stimulation of GH release	Divergent effects on food intake depending on species and administration sites. Stimulation of gut motility that indirectly stimulates food intake	(23, 28, 29, 56–61)
TRH	Growth	Stimulation of GH release	Stimulation of feed intake and locomotion	(23, 28, 29, 62)
CRH	Growth	Stimulation of GH release	Inhibition of food intake	(23, 28, 29, 63–65)
SS	Growth	Inhibition of GH release (basal and stimulated)	Role in metabolism Disparate effect on food intake depending on the administration site in mammals. No information for fish	(23, 28, 29, 66–73)
GH	Growth	Growth promotion Stimulation of IGF release in the liver and muscle	Orexigenic activity Induces the metabolism of protein, lipids, and carbohydrates	(23, 28, 29, 40, 74–82)
IGF-1	Growth	Stimulation of protein synthesis and turnover, cell proliferation. and differentiation	Role in peripheral metabolism and glucose homeostasis as shown in mammals	(23, 28, 29, 45–48)
Ghrelin	Food intake	Stimulation of food intake	Stimulate GH release in the pituitary	(28, 29, 83–89)
Nesfatin-1	Food intake	Inhibition of food intake Glucose homeostasis and lipid metabolism as shown in mammals	Inhibition of the GH–IGF system	(28, 29, 83, 90–98)
CCK	Food intake	Inhibition of food intake. Stimulation of pancreatic enzyme release, gallbladder contraction, and gut peristalsis	Stimulation of GH secretion	(28, 29, 83, 88, 99–102)
NPY	Food intake	Stimulation of food intake	Stimulation of GH secretion	(28, 29, 83, 88, 101, 103–106)
GRP	Food intake	Inhibition of food intake	Stimulation of GH secretion	(28, 29, 83, 84, 88, 99–101, 107)

The regulation of feeding behavior is based in the ventral tuberal hypothalamus where several brain nuclei produce either feeding-stimulating (orexigenic) peptides, particularly, agouti-related protein (AgRP) and neuropeptide Y (NPY), or feeding-inhibiting (anorexigenic) peptides, mainly melanocortin- stimulating hormone (aMSH), a pro-opiomelanocortin (POMC) derived peptide, and cocaine and amphetamine- related transcript (CART), which act in a coordinated manner to maintain food intake levels according to the requirements. As has been extensively studied in mammalian models, the primary hypothalamic nuclei involved in the regulation of food intake are the arcuate and the paraventricular nuclei (108). Teleost fish present the same neuropeptide control of food intake as mammals and have equivalent brain areas involved in it [for review, see (28, 29, 74, 83, 103, 108)].

The neuroendocrine control of the GH/IGF axis includes several stimulatory and inhibitory factors [for review, see (28, 29)]. The main stimulatory factors are the growth hormone-releasing hormone (GHRH) (109, 110) and pituitary adenyl cyclase-activating polypeptide (PACAP) (109, 111–114). In addition, other hypothalamic or gastrointestinal peptides and catecholamines also stimulate GH secretion. Among them that are worthy to mention are gonadotropin-releasing hormone (GnRH) (115–123), neuropeptide Y (NPY) (104–106), corticotropin-releasing hormone (CRH) (124), thyrotropin-releasing hormone (TRH) (125–127), ghrelin (84–87, 128), cholecystokinin (CCK) (84, 99, 100), gastrin-releasing peptide (GRP) (84, 99, 100, 107), nesfatin-1 (90, 129, 130), and dopamine (DA) (123, 131–135).

On the other hand, somatostatin (SS) is the main hypothalamic factor that inhibits both basal and stimulated GH secretion (112, 120, 123, 124, 136–141). In addition, norepinephrine inhibits GH secretion as well (142).

Several of the abovementioned neuropeptides regulating GH secretion are also involved in the control of feeding and energy balance (**Table 1**). For example, neuropeptides such as NPY, PACAP, SS, GnRH, TRH, and CRH and gastrointestinal peptides like CCK, ghrelin, GRP, and nesfatin-1, among others, have a role in feeding and energy balance regulation [for review, see (28, 29, 83, 88, 89, 103, 143, 144)].

This intricate regulatory network with many points of contact suggests a strong interrelationship between these two physiological processes. Furthermore, the GH itself, IGFs, and their regulatory network are involved in the regulation of feeding, energy balance, and metabolism [for review, see (53, 74)].

## 3 External regulation of endocrine factors controlling growth and food intake in fish

As mentioned earlier, the somatotropic axis is highly coordinated with the regulation of food intake as well as the reproductive endocrine axis. Thus, the somatotropic axis involves a great number of controlling factors briefly summarized in the previous sections. Therefore, the somatic endocrine axis would be highly informative in a wide range of growth-disturbing and stressful stimuli, and its study would represent a tool for assessing nutritional and environmental limitations (39). In this context, we will present the available information about abiotic factors affecting growth and the somatic endocrine axis.

### 3.1 Photoperiod

Photoperiod has been shown to alter somatic growth as well as the synthesis, secretion, and responsiveness of growth-related hormones (145–148). As a general plot, increasing light duration conducts to a higher growth rate and increased plasma IGF-1 levels (146, 149). Nonetheless, growth shows seasonal variation being increased during the summer and spring with non-parallel variations among GH and IGF serum levels and growth rates (149–155). For instance, a longer photoperiod results in higher GH levels or expression in Atlantic salmon (*Salmo salar*) (150, 152, 156), gilthead sea bream (*Sparus aurata*) (157), goldfish (*Carassius auratus*) (138), and coho salmon (*Oncorhynchus kisutch*) (158). Moreover, studies on Atlantic salmon suggest that the photoperiod may modify the effects of temperature on GH levels (159, 160). Thus, temperature and photoperiod should not be considered as independent controlling factors of fish endocrine growth axis (146). Furthermore, the individual effects of the two factors are difficult to dissect because fish are subjected to seasonal cycles and both photoperiod and temperature vary under natural conditions. Additionally, the relationship among GH, IGFs, and growth rates is affected by a wide range of endogenous and exogenous factors, such as gender, developmental and maturity state, nutritional state, temperature, salinity, and stress, in addition to photoperiod (39, 161, 162). Moreover, several of these factors, particularly those related to reproduction and feeding, are also affected by photoperiod and temperature.

### 3.2 Temperature

Fish, as aquatic ectotherms, must be able to contend with continuous fluctuations in water temperature, which, in turn, influences gene expression and the activity of metabolic enzymes (163–167). In fact, environmental temperature is one of the most important ecological factors, which influences the behavior and physiological processes of aquatic animals (145, 168).

Seasonal variations of somatic growth as well as in GH levels are associated with water temperature changes. Thus, it has been shown that pituitary GH content or plasma GH levels and also IGF-1 levels were increased during spring and summer in several teleost species such as perch (*Perca fluviatilis*) (169), brown bullhead (*Ictalurus nebulosus*) (170), coho salmon (171), goldfish (138), rainbow trout (*Oncorhynchus mykiss*) (172), gilthead sea bream (157, 173), chinook salmon (*Oncorhynchus tshawytscha*) (174), and common carp (*Cyprinus carpio*) (175). Consequently, it is likely that water temperature regulates the plasma levels of GH and IGF-1.

Although GH has been detected at the early stages of embryo development and post-hatching (176–178), it seems that water temperature does not affect GH cell differentiation or GH expression (179, 180) at these early stages of life. Even so, the expression levels of IGF-2 and IGF-1 receptors were upregulated when incubated in elevated temperatures in correlation with the enhanced embryonic growth rate found in this condition (181). However, at hatching, the expression levels of IGF-1 and IGF-2 were higher at the lowest temperature (4°C) (179, 181). These findings indicate that embryonic growth is GH independent and there is a shift in growth regulation and temperature affects it upon hatching.

The effect of temperature, independent of photoperiod, on GH serum levels can be experimentally addressed. For example, when Nile tilapia, *Oreochromis niloticus*, was maintained in increased temperature, higher circulating GH levels were observed (182). Rainbow trout reared at increasing temperatures shows that growth rate correlates with temperature (183). In this experiment, higher temperatures increased IGF-1 plasma levels and hepatic IGF-1 mRNA levels (183) stimulated by GH. Increasing water temperature during winter (short photoperiod) stimulates growth rates and increases plasma GH levels in sea bream. Conversely, decreasing water temperature during summer (long photoperiod) decreases growth rates and circulating GH levels (138, 157). Thus, it seems that temperature, independent of photoperiod, induces higher GH and IGF secretion, which positively correlates with increased growth rates (184).

However, the positive correlation between increased temperature and increased plasma GH could not translate into enhanced fish growth because of stress or undernutrition. For instance, Atlantic salmon reared at increasing temperatures shows a biphasic growth rate increasing from 4.6°C to 14.4°C and decreasing between 14.4°C and 18.9°C while reaching the highest amounts of plasma GH at 18.9°C (185). Moreover, increasing water temperature close to the maximum of the tolerable range elevates GH levels regardless of feed conditions (166, 183, 186). However, IGF-1 concomitantly increases only in *ad libitum*-fed fish, while fish-restricted feeding regimens showed reduced IGF-1 levels and reduced growth rates (183). It can be concluded that fish under *ad libitum* feeding regimen had an increase in GH which is translated into an increase in IGF-1 levels and somatic growth regardless of the rearing temperature, while fish under restricted feeding and high temperature exhibited a reduced GH receptors’ response, with reduced IGF-1 and growth rates and increased GH levels which may reflect a GH resistance (see discussion in Section 3.3 below). Thus, the positive effect of temperature on hepatic IGF-1 synthesis was avoided (166, 183). Therefore, nutritional condition interferes with the somatic growth endocrine axis. Additionally, higher temperatures increase feed consumption (149, 166), which indeed stimulated somatic growth (145).

### 3.3 Nutrition

Nutrient and diet composition strongly influence somatic growth in fish (28, 187). The synthesis and release of hepatic IGF-1 are largely affected by food restriction or deprivation as shown by studies in several salmonid species (166, 183, 188–191) as well as in gilthead sea bream (192, 193) and hybrid striped bass (*Morone chrysops* x *Morone saxatilis*) (194, 195). Given that GH stimulates lipolysis independently of IGFs and growth promotion, it plays an important role in energy mobilization in the context of undernutrition or nutrient deficiency. Thus, elevated circulating levels of GH along with low IGF-1 confer a metabolic advantage under this condition (39). Therefore, a state of GH resistance, a reduced hepatic responsiveness to the anabolic GH action, is highly conserved through the evolution of fishes and higher vertebrates (39). The GH resistance may be the result of reduced GH receptor expression or post-receptor defects in GH signaling (39). In this context, it was shown that the hepatic transcript of *ghr-1* correlates with changes in growth rates, plasma IGF-1 level, and hepatic *igf-1* expression (39, 196–198). In addition, deficiencies of nutrients as a consequence of unbalanced diets downregulate hepatic *ghr-1* expression (197–199), indicating the prominent role of GHR-1 in the systemic growth-promoting action of GH.

It has been shown that, in teleost, nutrients like glucose, amino acids, and lipids influence hormones and peptides that regulate feeding such as nesfatin-1, ghrelin, CCK, and leptin, as well as the GH/IGF axis [for review, see (28)]. The way nutrients influence feeding growth and metabolism is not fully understood, but it would depend on nutrient- sensing mechanisms located in several tissues such as the gut, liver, pancreas, and hypothalamus (200–202). These nutrient- sensing mechanisms are capable of determining directly or indirectly the levels of nutrients and stored energy [for review, see (28, 103)]. Thus, nutrients in the luminal surface of the gastrointestinal tract (GIT) evoke changes in the expression and activity of digestive enzymes and the synthesis and secretion of GIT peptides and hormones such as CCK, GLP-1, GIP, PYY, secretin, ghrelin, or nesfatin-1 (28). Therefore, the GIT conveys information about the type and levels of nutrients through the vagal enteric afferents and/or an endocrine pathway to the brain as shown for mammalian species (203). Furthermore, circulating levels of nutrients modulate the responses of hypothalamic neurons that express either AgRP and NPY (AgRP/NPY neurons) or CART and POMC (CART/POMC neurons). As a general pattern, higher nutrient levels activate the CART/POMC neurons and inhibit the AgRP/NPY neurons (201) inducing satiation. Although it was described that these peptides affect the GH/IGF axis (29, 83), it is not clear at this point if GH is concomitantly affected. Nevertheless, nutrients such as proteins, amino acids, and lipids modulate the GH/IGF axis [for review, see (28)]. For instance, it was shown in rainbow trout that in well-fed fish, GH induced hepatic IGF expression through a signaling pathway involving JAK-STAT, PI3K-Akt, and ERK. However, under fasting conditions, GH stimulated lipolysis through PKC/phospholipase C (PLC) and MAPK/ERK pathways (40). Furthermore, two GH receptor (GHR) isoforms have been described in teleost fish named GHR-I and GHR-II with additional variants in salmonids (GHR2a, GHR2b) (40, 204, 205). If the receptor isoforms are involved in switching signal transductions is not yet established. Nevertheless, according to tissue expression patterns and changes in the expression levels of the isoforms depending on nutritional status, it is suggested that GHR-I could be involved in growth promotion, while GHR-II could be involved in the metabolic response to GH (40). However, experimental confirmation is still required. Thus, nutrient availability is sensed, and a coordinated response by the central and peripheral systems is executed to provoke either somatic growth or internal maintenance processes.

### 3.4 Salinity

The relationship between the somatotrophic axis and osmoregulation has been extensively demonstrated (206–211). In this regard, GH and IGF- 1 improve acclimation and survival to high salinity water (212–220). Conversely, water salinity modulates growth rates, food intake, and feed efficiency ratio in several species. When euryhaline or freshwater fish species were transferred to high salinity water, they showed a higher growth rate and improved feed efficiency ratio (221–225). In marine fish species, however, intermedia salinities improve growth rates and feed conversion (226–230). The mechanisms by which water salinity influences growth are not fully understood; although, a reduction of energy expenditure related to osmotic regulation would be involved (207). Additionally, increasing water salinity stimulates GH and IGF production. For example, plasma GH and IGF- 1 levels were increased in catfish and trout when transferred to high salinities (231, 232). Moreover, changes in water salinity modulate the expression levels of genes of the somatic growth axis in salmonids (213), blackhead sea bream (*Acanthopagrus schlegelii*) (233), gilthead sea bream (234), tilapia (*Oreochromis mossambicus*) (235), and pejerrey (*Odontesthes bonariensis*) (236). Furthermore, high salinity increases food intake (237, 238), perhaps stimulated directly by GH or indirectly through the metabolic effects of GH (75, 239, 240). Moreover, GH improves nutrient absorption at the gut level (241) which can explain the observed increase in feed efficiency.

### 3.5 Oxygen

The concentration of dissolved oxygen (DO) in water would affect the performance of fish as oxygen is required for respiration and metabolism. Environmental conditions regarding oxygen partial pressure, water temperature, and salinity as well as oxygen consumption by aquatic organisms (biological oxygen demand) would affect the amount of DO in the water column (242). Low levels of DO or hypoxia could be defined as the values of DO that negatively affect the physiology or behavior of fish (242–245). Hypoxia conditions reduce respiration, feeding activities, and growth rate and increase the chances of disease in fish (243, 246–248). Furthermore, hypoxia induces behavioral, physiological, immunological, and metabolic adjustment [for review, see (243)] including swimming behavior, declined metabolic rate, high ventilation and anaerobic respiration, and high Hb-O_2_ affinity to cope with low oxygen stress. In addition, hypoxia inhibits gonadal development and reproduction as was shown in goldfish exposed to different diel hypoxia cycles (249). Furthermore, hypoxia provokes a reduction in egg production and spawning, delays embryo development, and increases embryo and larvae mortality (242).

In terms of somatic growth and feeding, it has been shown that low DO level negatively impacts growth, feed intake, and overall fish performance (242, 244, 245). In Nile tilapia reared at high, medium, and low DO, the fastest rate of growth was at high DO and the slowest growth was at low DO (250). In the same study, the authors found that the food conversion ratio (FCR) was inversely related to the dissolved oxygen level. Thus, exposure to hypoxia reduced growth and feed utilization, as well as respiration and feeding activities (251–253). It has been shown in several fish species that hypoxia inhibited weight gain, probably due to low feed intake (252, 254–260).

Reduced food intake at low DO has been found in several fish species such as channel catfish (*Ictalurus punctatus*) (261, 262), rainbow trout (263), blue tilapia (*Oreochromis aureus*) (264), European sea bass (*Dicentrarchus labrax*) (265), juvenile turbot (*Scophthalmus maximus*) (252), and Nile tilapia (250, 257–260).

Nevertheless, how hypoxic conditions affect the regulatory endocrine axis for both somatic growth and feeding is not clear. Somatic growth inhibition and lowered plasma IGF-1 occur in hypoxic conditions as well as in crowded fish (266, 267). Thus, activation of the stress response of the hypothalamus–pituitary–interrenal (HPI) axis could be responsible for the inhibition of hepatic response to GH (266, 267). However, *gh*-transgenic fishes show a reduced capacity to cope with a hypoxic environment (268, 269), suggesting some interaction between hypoxia and the GH/IGF axis. In this regard, it was shown that knocking down *insulin-like growth factor binding protein* (*igfbp*)*-1*, a known IGF- binding protein with growth inhibition effect (270–272), moderates the hypoxia effects on growth and development in zebrafish (*Danio rerio*) (273). On the contrary, overexpression of *igfbp-1* mimics hypoxia- induced growth and developmental retardation, even under normoxia (273). Furthermore, sirtuins, which are involved in conveying energy level information and modulating the anabolic action of GH by inhibiting GH receptor signaling (28, 39), could act as links between oxygen availability, energy status, and the somatotropic axis (39).

## 4 Effect of different pollutants on the fish endocrine network governing growth and feeding

Fish are particularly vulnerable to pollution because they are heavily exposed to contaminants as they feed and live in the aquatic environment. Therefore, fish are more sensitive to many toxicants in comparison with other vertebrates, making them an important subject of experimentation and good bioindicators of ecosystem health. In this section, we will cover the knowledge about the impact of pollutants on the endocrine axes that regulate growth and food intake behavior in fish.

A key role in the integration of stress response is played by the steroid hormone cortisol. Cortisol is usually released into circulation when fish are under conditions of stress by the activation of the vertebrate hypothalamus–pituitary–adrenal (HPA) axis, so- called the “stress axis.” Cortisol is one of the downstream main effectors of the HPA axis, playing essential roles in development, energy balance, and behavior (274). One notorious effect of cortisol is an increase in the plasmatic levels of GH (217, 275, 276). Aside from a possible direct effect at the pituitary level increasing GH synthesis and release (277), cortisol decreases the hepatic expression of the GH receptors *ghr1* and *ghr2* and decreases the serum levels of IGF-1 and the liver expression of *igf-1* (162). Thus, corticosteroids could induce a state of “GH resistance” characterized by high serum GH levels concomitant to low serum IGF- 1 levels. Moreover, in chronic treatments, cortisol led to a reduction of somatic growth in fish. Concerning food intake regulation, it has been reported that chronic and acute stress treatments exert a potent anorexigenic effect in fish involving several hypothalamic neuropeptide s such as CRH and aMSH, along with an increase in cortisol levels (278). This steroid hormone seems also to be involved in the maintenance of the anorectic response in teleost (279–281). The synthesis and release of cortisol in response to contaminants in fish have been very well documented. However, the specific connection between each pollutant and the expression of factors governing growth and or food intake, mediated by cortisol, is not always evaluated. Therefore, in those cases in which the effect of pollutants on cortisol as mediating their effects on growth and food intake was not studied, we will only mention it to point out the need for additional studies.

### 4.1 Heavy metals

Heavy metal pollution in aquatic environments is the result of anthropogenic and natural activities, such as atmospheric deposition, geological weathering, and the discharge of agricultural, municipal, residential, or industrial waste products. When heavy metals occur in high concentrations, they are a serious threat because of their toxicity, long persistence, bioaccumulation, and biomagnification in the food chain. Low levels of pollution may have no apparent impact on fish, but it may decrease the fecundity of fish populations, leading to a long-term decline and eventual extinction of species (282). Heavy metals negatively affect various metabolic processes in fish embryos, resulting in developmental retardation, morphological and functional anomalies, and ultimately premature death. Additionally, heavy metals activate energy-consuming detoxification processes resulting in less energy available for growth (283). Cortisol is usually released when fish are under conditions of stress, and one of the consequences observed is an increase in the plasmatic levels of growth hormone and a concomitant reduction in IGF levels (217, 275, 276). In chronic treatments, this led to a reduction of somatic growth.

Concerning the impact of heavy metals at the endocrine level, it was found that cadmium delays the expression of GH during the early development of rainbow trout, demonstrating an endocrine-disrupting capacity *in vivo* of cadmium in teleost fish (284). Lead was also found to inhibit the expression of GH in roho labeo (*Labeo rohita*). Concentrations of Pb from around the LC_50_ were found to reduce the growth and consistently the plasmatic levels of GH while increasing cortisol, compared with the control group during 5 weeks (285). When Prussian carp (*Carassius auratus gibelio*) were exposed to Cd at 1, 2, and 4 mg/l per 30 days, the gene expression levels of several neuropeptides in the brain were consistent with the observed changes in food intake behavior (286). In all the Cd-exposed groups, the expression levels of NPY, apelin, and metallothionein increased significantly, while those of POMC, ghrelin, and CRH decreased significantly. The authors suggested that low doses of Cd might increase food intake, as well as weight and length gains, but high doses of Cd might have the opposite effect. The observed changes in food intake seem to be a result of neurohumoral regulation in response to Cd exposure. The expression levels of three genes (*gh*, *ghr*, and *igf-1*) related to the growth endocrine axis were shown to be altered in grass carp (*Ctenopharyngodon idella*) under mercury chloride (HgCl_2_) and temperature stress. According to the authors, this result indicated that the combined stress of temperature and HgCl_2_ affected the growth performance of grass carp at the molecular level. In this study, the expression of *gh* and *ghr* has increased, while that of *igf-1* has decreased, indicating that there was a subtle interaction between the two experimental factors (287). These results show that the binding ability of GH and its receptor decreases in fish under physiological stress, resulting in the decrease of signal from GHR, leading to the inhibition of IGF-1 synthesis and ultimately decreasing fish growth (236, 288). Arsenic is a contaminant commonly present in water and crops from several parts of the world, and exposure to arsenic is linked to decreased birth weight, decreased weight gain, and improper muscle function. The effects of embryonic arsenic exposure on muscle growth and on the IGF pathway were tested in killifish (*Fundulus heteroclitus*) by Szymkowicz et al. (289). The authors found significant reductions in condition factors of fish exposed to arsenic after 16, 28, and 40 weeks. Additionally, they found that exposure of killifish embryos to arsenic has long-term effects, reducing growth and increasing both *igf-1* and *igf-1r* levels in skeletal muscle even after 1 year. Since fish presented reduced growth, the higher expression levels of *igf-1* and its receptor in skeletal muscle might be related to a dysregulation in their expression originated by the early exposition to As^III^. In common carp, a decreased expression of *gh* mRNA was detected in the pituitary in response to Zn (290). In rainbow trout, a decreased expression of *gh*, *igf-1*, and *igf- 2* was detected in an exposure time-dependent response to Zn and Co (291). The authors conclude that *igf- 1* is the most resistant and *gh* is the most sensitive component against cobalt and zinc exposure. The effect of Zn and Cu on the action of CCK on the enzymes peptidases and glycosidases in goldfish intestine was examined. Interestingly, both metals affect the activity of glycosidases induced by CCK. This means that the action of CCK is influenced by the presence of Zn and Cu, which could affect other processes involving the action of CCK (apart from nutrient assimilation) such as food intake regulation (292).

The effect of a mixture of heavy metals was studied in Nile tilapia (293). Adults chronically exposed (5 weeks) to a mixture of Pb, Cu, and Zn at sublethal concentrations showed a decrease in all parameters related to growth and body weight compared with control. Consistently, the expression of *igf-1* and *ghrelin* in liver and stomach, respectively, decreased in the exposed group compared with the control group (293).

### 4.2 Persistent organic pollutants

Persistent organic pollutants (POPs) are ubiquitous environmental contaminants that are not easily degraded and can biomagnify in aquatic and marine food webs (294). The list of POPs includes a wide range of halogenated contaminants such as polychlorinated biphenyls (PCBs), dichlorodiphenyltrichloroethane (DDT), chlordanes, and hexachlorobenzene (HCB) as well as chemicals of emerging concern such as polybrominated diphenyl ethers (PBDEs) or perfluorinated compounds. Because of their lipophilic or proteophilic nature, they can be found at high concentrations in the tissues of aquatic organisms (295, 296). POPs could originate as industrial compounds or pesticides, being produced during natural disasters (e.g., forest fires, volcanic eruptions), as a by-product during wood pulp processing or in the synthesis of chlorinated chemicals, or as a result of incineration of chlorine-containing compounds (297). Although POPs have relatively low hormonal activity, they are persistent in animal tissues and, thus, pose a serious risk as endocrine disruptors. There is a wide range of molecular interactions between POPs and the endocrine system of fish. The effects can vary depending on the chemical, the fish species, sex, reproductive stage, and exposure conditions, among other factors. The action of POPs as endocrine disruptors could be at different levels, such as hormone synthesis, transport and secretion, transformation, excretion or clearance, receptor recognition/binding, and post-receptor response (298). Thus, the mechanisms of POP-induced toxicity at the endocrine level can be complex, with limited information on the molecular interaction between these toxicants and the hormonal system. In zebrafish, the developmental effects of lifelong exposure to environmentally relevant concentrations of two natural mixtures of POP s were investigated by Lyche and colleagues (299). The mixtures emulate those found in freshwater systems in Norway, with high and background levels of PBDE s, PCB s, and DDT metabolites. The phenotypic effects observed in both exposure groups included differences in body weight at 5 months of age. Genome-wide transcription profiling showed changes in the regulation of genes involved in endocrine signaling and growth. Consistent with the changes observed at the growth level, the transcriptomic changes include key regulator genes for steroid hormone functions (*ncoa3*) and growth (*c/ebp*, *ncoa3*) (299).

Although POPs have been highlighted as endocrine disruptors of special concern, as far as we know, their impact has been studied on the thyroid and reproductive axes (300). Only the abovementioned study reports an effect on the growth endocrine axis, highlighting a clear gap of knowledge since it was widely demonstrated that POPs generated several alterations of growth in fish (301). Additionally, and to the best of our knowledge, no studies have been carried out to cover the effects of POPs on the endocrine regulation of food intake in fish.

### 4.3 Agrochemicals

The existence of several agrochemicals, popularly known as pesticides, in water bodies such as dams, lakes, streams, and rivers generates a multifarious exposure of these chemicals to aquatic organisms (302). Pesticides are known as elements released into the environment to kill fungi, weeds, insects, rodents, etc. Despite their advantages for the human economy, pesticides might have harmful effects on humans and other organisms (303). Based on their harmfulness, the World Health Organization (WHO) grouped them into four categories, namely, exceedingly hazardous, greatly hazardous, abstemiously dangerous, and considerably harmful (304). Although there are a few reports indicating the effects of agrochemicals on growth and metabolism in fish (305–307), there are very few reports on the effect on the endocrine growth axis. A possible alteration of the growth endocrine axis by agrochemicals was found in a study by Singh and colleagues (308). In this study, high levels of pesticides (hexachlorocyclohexane, dichlorodiphenyl-trichloroethane, and chlorpyrifos) were co-related with low levels of cortisol in wild populations of catfish. It is inferred that low levels of cortisol lead to an increase in the expression of GH, but the real effect on fish growth was not determined. The effect of two widely used insecticides, chlorpyrifos and esfenvalerate, was evaluated on the expression of *igf-1* in juveniles of Chinook salmon. A decreased expression of *igf-1* was found in the spleen and related by the authors to immune system response and growth effects (309). Clearly, there is a knowledge gap about the impact of agrochemicals on the endocrine growth axis and the endocrine factors regulating food intake in fish. In the search for molecular biomarkers suitable for use in aquatic toxicology, knowing the effect of agrochemicals on the expression of genes of the abovementioned axes would be a great advantage.

### 4.4 Pharmaceutical and personal care products

Pharmaceutical and personal care products (PPCPs) are widely used by individuals for health or cosmetic reasons or by industries to promote growth or protect the health of production animals. These chemicals include a huge variety of therapeutic and veterinary drugs, fragrances, and cosmetics. The number of PPCPs has increased exponentially during the past decades, with most of them being synthetic compounds. Therefore, fish do not have a completely evolved tolerance to these chemicals. Thus, the large number of PPCPs and their diversity in chemical structures and toxicology currently pose a significant challenge in understanding the adverse environmental and health impacts of these products on animals and humans. Moreover, in the environment, organisms are exposed to mixtures of chemicals which exert their effects through complex chemical, toxicological, and physiological interactions (310). Therefore, in this section, we will describe as far as possible the interactions between PPCPs with the endocrine growth axis and hormones governing food intake behavior.

The effect of environmental estrogens (EEs) such as 17β-estradiol (E2), β-sitosterol (βS), and 4-n-nonylphenol (NP) on the synthesis of IGFs on rainbow trout was studied by Hanson et al. (311). The authors found that E2, βS, and NP significantly inhibited the expression of *igf-1* and *igf-2* mRNAs in the liver and gill in a time- and concentration-related manner. The mechanism through which EEs inhibit *igfs* mRNA expression was investigated *in vitro* in isolated liver cells. The authors determined that EE treatment deactivated JAK, STAT, ERK, and AKT. Also, the blockade of GH-stimulated *igf* expression by EEs was accompanied by the deactivation of JAK, STAT, ERK, and AKT. Overall, the results indicate that EEs directly inhibit the expression of igf mRNAs by disrupting GH post-receptor signaling pathways (311). Embryos of rainbow trout were exposed to graded concentrations of E2 (measured: 0, 1.13, 1.57, 6.22, 16.3, 55.1, and 169 ng/L) from hatching to 4 and up to 60 days post-hatch (dph) to assess molecular and apical responses (312). Whole proteome analyses of alevins did not show clear estrogenic effects, but among other results, some terms were overrepresented, including regulation of IGF transport and uptake by IGFBPs, apolipoprotein A1/A4/E domain, and lipid transport. Although all these factors have been previously associated (by other authors) with endocrine functions such as growth, this apical response was not observed in this work. The effects of 17a-ethinylestradiol (EE2), 17β-estradiol, and 4-nonylphenol on *igfbps* were studied in two stages of Atlantic salmon by Breves et al. (313). In fry and smolts, hepatic *ghr*, *igf-1*, and *igf-2* were diminished by exposure to endocrine disruptor chemicals (EDCs). In fry, EDCs diminished *igfbp1b1*, *igfbp2a*, *igfbp2b1*, *igfbp4*, *igfbp5b2*, and *igfbp6b1*. In smolts, EDCs diminished *igfbp1b1*, *igfbp4*, and *igfbp6b1*. *Igfbp5a* was stimulated by EDCs in both fry and smolts. The authors conclude that hepatic *igfbps* directly or indirectly respond to environmental estrogens during two key life stages of Atlantic salmon and, thus, might modulate the growth and development of exposed individuals. Shved and coworkers (314) investigated whether estrogen exposure during early development affects growth and the IGF- 1 system, both at the systemic and tissue levels in Nile tilapia. Developmental exposure to 17a-ethinylestradiol had persistent effects on sex ratio and growth. Serum IGF-1, hepatic *igf-1* mRNA, and the number of *igf-1* mRNA-containing hepatocytes were significantly decreased at 75 days post-fertilization (DPF), while liver receptor alpha mRNA was significantly induced. In both sexes, pituitary GH mRNA was significantly suppressed. A transient downregulation of *igf-1* mRNA occurred in the ovaries (75 DPF) and testes (90 DPF). These results clearly show that estrogen treatment impairs GH/IGF- 1 expression in fish and also that the effects persist during development. These long-lasting effects seem to be exerted indirectly *via* inhibition of pituitary GH and directly by suppression of local IGF-1 in organ-specific cells (314). These authors also demonstrated in a related work that environmentally relevant concentrations of EE2 interfere with the GH/IGF- 1 system, at the endocrine, paracrine, and autocrine levels in developing Nile tilapia (315). These results at the molecular level were also accompanied by diminished growth and weight gain in male fish. All these results demonstrated that estrogens released into the environment interact with several endocrine functions in fish, related not only to reproduction but also to growth and metabolism.

The influence of PPCPs on endocrine factors regulating food intake in fish was not yet determined. This is an interesting topic to address particularly in the case of EEs since they can affect the intrinsic relationship between reproduction, growth, and food intake in fish (23, 316, 317). As an antecedent, a study carried out by Bertucci et al. (318) aimed to determine whether estradiol (E2) and testosterone (T) affect the expression of several components of this axis: preproghrelin, ghrelin/growth hormone secretagogue receptor (GHS-R), ghrelin O-acyl transferase (GOAT), and NUCB2 in goldfish. RT- qPCR analyses at 2.5 days post-administration show that gut preproghrelin and GOAT expression was upregulated by both E2 and T treatments, while the same effect was observed for GHS-R only in the pituitary. Both treatments also reduced hypothalamic preproghrelin mRNA expression. NUCB2 expression was increased in the forebrain of the T-treated group and reduced in the gut and pituitary under both treatments. These results showed modulation by sex steroids of genes implicated in both food intake behavior and reproduction that might help to explain the reproduction- dependent energy demands in fish and highlight a possible negative impact of PPCPs in wild fish.

### 4.5 Micro- and nano plastics

There are several works studying how microplastics and, to a lower extent, nanoplastics can affect fish growth [for review, see (319–321)]. Although most of these works were carried out on model fish species and with concentrations of plastic particles that are several orders of magnitude above the range found in the environment (322), they provide novelty data about the problem of plastics in the aquatic environment. A few of these articles studied the effect of micro- or nanoplastics on the endocrine growth axis. Some works investigated the effect of microplastics and their combinations with other pollutants at the transcriptomic level, but no reports of genes involved in growth were found (323–325). Zheng and colleagues (326) investigated the effects of embryo–larvae exposure to 500 μg/L of polystyrene microplastic (MP) (5 µm) alone and in combination with ZnO nanoparticles (NPs) on exposed F0 and unexposed F1 larvae of zebrafish. The authors found that growth inhibition, oxidative stress, apoptosis, and disturbance of the GH/IGF axis were induced by MPs alone in F0 larvae. Reduced growth and antioxidant capacity and downregulated GH/IGF axis were merely observed in F1 larvae from F0 parents exposed to MPs + ZnO. In another work carried out by Pedersen and colleagues (327), the early growth and development of zebrafish was not impacted by NP, but the endocrine system, disease rate, and organismal development were significantly altered by NP exposure.

Microplastics can be ingested by fish and accumulated in the stomach, generating a sensation of satiety or damage that could inhibit food intake. This was previously reported (328–331) although the effect of MP on factors that regulate food intake has not been deeply studied yet. We found only one report, by Im and coworkers (332), that investigated the neurodevelopmental toxicity of polystyrene microplastics (PSMPs) in zebrafish. Fish were exposed to PSMPs during the embryonic stage and then allowed to recover. Among other results, the authors reported an increased expression of NPY, a peptide involved among other functions in the increase of appetite. However, no relations with food intake were found in that work. On the other hand, the ingestion of polyethylene microplastics by larvae and juveniles of medaka (*Oryzias latipes*) does not affect the expression of molecular markers involved in glucose metabolism, energy homeostasis, weight gain, and proteolysis (ISN, GLP, PYY, and TRP) (333).

As a conclusion, there are few reports indicating that MP or NP can trigger a stress response inducing mechanisms that involved the GH–IGF axis. Moreover, their very well-documented accumulation in the stomach of fish might alter the response of peripheral factors regulating food intake, but surprisingly, this has not been deeply studied yet. These two points represent a knowledge gap that would be interesting to address to do a proper estimation of the potential risk of MP and NP present in the marine environment.

## 5 Effects of global climate change factors on growth and feeding endocrine axis

Fish are severely exposed to all changes produced by global climate change that currently impacts the oceans. There are predictions that indicate an increase in the mean values of water temperature, an increase in dissolved CO_2_ with the concomitant decrease in water pH, and higher variations in salinity, among others (334). In this section, we will summarize the data on the effects of ocean acidification and ocean warming on fish endocrine axis regulating growth and food intake.

### 5.1 Ocean acidification

During the last 150 years, the burning of fossil fuels has contributed to an increase in atmospheric CO_2_ from approximately 280 to 410 ppm, with a predicted increase of 730 to 1,020 ppm by the end of 2100 (335). The absorption of atmospheric CO_2_ by oceans generates rapid changes in the seawater carbonate system ultimately decreasing the pH (among other effects), a process termed ocean acidification (OA). Exposure to these conditions can severely impact marine organisms as the acclimation to a suboptimal environment requires physiological adaptive responses that are energetically costly. Consequently, fish experience environmental stress, which may impact them at the biological and physiological levels (336). A comparison of the effects of OA on the growth and development of the early life stages of marine fish indicates that these are species-specific, and thus, generalizing the impact of climate-driven ocean acidification is not warranted. The effect will ultimately depend on the capacity of the specie to balance the energy costs of acid–base homeostasis *versus* that contributing to somatic growth.

An interesting case study that related growth with food intake was carried out in black sea bream by Tegomo et al. (337). Although the authors did not analyze the endocrine response of the growth axis, they observed that under two projected scenarios of OA, with pH values of 7.80-pCO_2_ = 749.12 ± 27.03 and 7.40-pCO_2_ = 321.37 ± 11.48 μatm, the growth, feeding efficiency, protein efficiency ratio, and crude protein content were significantly decreased in juveniles. The authors conclude that, because of an elevated pCO_2_ in seawater, the fish eat more than normal but grow less than normal. These results evidence that ocean acidification provokes changes at the endocrine level inducing an increase in feeding behavior while generating diminished growth. Interestingly, Carney Almroth et al. (338) found that the enzyme BChE was decreased at elevated CO_2_ treatment in the Atlantic halibut, *Hippoglossus hippoglossus*. The BChE enzyme was inversely correlated with circulating levels of the orexigenic peptide ghrelin in mice (339). Therefore, these decreases in BChE levels under OA conditions could explain the endocrine source of the increase in food intake observed by Tegomo et al. (337) in black sea bream.

A study of the complete transcriptomic response in Asian sea bass (*Lates calcarifer*) juveniles reared for 7 days under OA conditions showed tissue-specific differentially expressed genes (340) involving many molecular processes in the brain, gill, and kidney. The results indicate that although short-term ocean acidification does not cause obvious phenotypic and behavioral changes, it causes substantial changes in gene expression in the analyzed tissues. These changes are related to organ and muscle development, growth, and nervous system development, as well as behavior related to memory (e.g., hunting, homing, and escaping from predators). The authors conclude that these changes in gene expressions may eventually affect the physiological fitness of fish (340).

In the Atlantic salmon, the physiological adaptations which take place during the early phase of salmon ocean migration (high hypo-osmoregulatory capacity, growth, and energy metabolism) are all processes that are under the control of the growth endocrine axis (341). The osmoregulatory development that allows smolts to have high seawater tolerance while still in freshwater may make them more susceptible to external stressors, particularly those that affect ion regulation, such as water acidification. The smolt stage of Atlantic salmon is then particularly sensitive to acidification, and it was observed that moderate conditions can lead to increased mortality and less severe conditions result in loss of salinity tolerance and reduced adult return rates (342).

Shao et al. (343) found that OA suppresses the mRNA expression of insulin-like growth factor 1 and reduces the growth rate in juveniles of orange-spotted groupers (*Epinephelus coioides*). In this study, the authors exposed fish to different levels of acidification: a condition predicted by the Intergovernmental Panel on Climate Change (IPCC, pH 7.8 - 8.0) and an extreme condition (pH 7.4 - 7.6) that may occur in coastal waters in the near future. After 6 weeks, the growth rates of fish reared at pH 7.4 - 7.6 were less than those raised in control water (pH 8.1 - 8.3). Additionally, exposure to pH 7.4 - 7.6 resulted in lower levels of hepatic *igf-1* mRNA but did not affect the levels of pituitary *gh* or hypothalamic pre-pro-somatostatin II and III (*psst2* and *psst3*, respectively). Overall, these results show that ocean acidification conditions suppress the growth of juvenile grouper, which may be a consequence of reduced levels of IGF-1, but not due to diminished growth hormone release (343). To test the effects of climate change on juvenile blue rockfish (*Sebastes mystinus*), Bruzzio (344) measured the endocrine response to single and combined stressors of OA and hypoxia after 1 week of exposure. Assays of cortisol and IGF-1 hormone responses served as proxies for stress and growth, respectively. There was no observable difference in IGF-1 in juvenile blue rockfish after a week of exposure to stressors such as low pH or low DO. The author also found a high peak of cortisol with low pH presumably associated with the role of cortisol in acid–base regulation. Interestingly, when cortisol levels were high, the same fish had low levels of IGF-1, but when cortisol levels were lower, the same fish had highly variable levels of IGF-1. Overall, the results indicated that pH levels influenced hormonal stress physiology, while DO levels contributed to differences in metabolism, body condition, and behavioral anxiety in juvenile blue rockfish.

All these reports highlight the necessity to investigate the effects of climate change on fish endocrine response related to growth and food intake behavior to find interrelationships with physiological and biological effects. It will be important to obtain more knowledge on molecular markers that can be used for fish population management in a rapidly changing ocean.

### 5.2 Ocean warming

The ocean absorbs most of the excess heat from greenhouse gas emissions, leading to rising ocean temperatures in a phenomenon known as ocean warming (OW). Increasing temperatures affect marine species causing a stunning impact on ecosystems, such as coral bleaching and the loss of breeding grounds for marine fishes and mammals. The effects of OW are being widely studied in marine invertebrates although not much information is available for fish, neither at the biological nor at the endocrinological level. At a physiological level, temperature has been proven to affect metabolic rate, aerobic metabolism, growth, reproduction, and survival with potential effects on population sustainability (345). Moreover, changes at the individual level are dependent on the capacity to modulate gene expression under environmental variation (346, 347). Changes in cellular stress proteins (heat shock proteins, antioxidant enzymes, etc.) have been detected in marine organisms exposed to acute and chronic heat stress (348, 349). Nevertheless, these studies have only focused on a few protein biomarkers and therefore do not explore all the possible changes caused by OW. In this section, we will summarize the knowledge about the influence of environmental temperature increase on the endocrine growth axis and, if available, on the endocrine regulation of feeding behavior.

The effect of water temperature increase seems to be specie-specific. For example, no changes in the mRNA expression of *igf-1* with the temperature increase were found in European sea bass after 15 and 60 days of exposure, but a decrease in the *myog* gene (associated with muscle growth) was found at 60 days (350). In European eel (*Anguilla Anguilla*), the impact of temperature on thermally induced phenotypic variability from larval hatch to first feeding was studied by Politis et al. (351). The results showed that increasing temperature from 16°C to 22°C accelerated larval development, while gene expression of *hsp70*, *hsp90*, *gh*, and *igf-1* was also accelerated at warm temperatures. On the contrary, larval gene expression patterns (*hsp70*, *hsp90*, *gh*, and *igf-1*) were delayed at cold temperatures. Moreover, the expression of *gh* was the highest at 16°C during the jaw/teeth formation and the first-feeding developmental stages. These results highlight the differences in the effect provoked by the increase of water temperature through developmental stages. In conclusion, the authors suggest a plausible vulnerability of this critically endangered species in future scenarios of increasing ocean temperature. The larvae of the gilthead sea bream exposed to three temperatures —18°C (control), 24°C (warm), and 30°C (heat wave)—for 7 days showed a survival decrease with increasing temperature, with no larvae surviving at 30°C. A proteomic analysis (that was only carried out at 18°C and 24°C) showed that larvae upregulated, among other proteins, the growth hormone. These results might indicate that although the temperature increase could generate a proteome modulation, gilthead sea bream larvae would not be able to fully acclimate to higher temperatures. This conclusion was supported by the observed low survival rates at high temperature (352).

Overall, as it was mentioned before, the effect of ocean warming seems to be dependent on the fish specie and on the developmental stage, leading to changes in larvae that could result in mortality or malformations altering the future stages of these fish. Independent of the specie, as mentioned in Section 3.2, temperature induces changes in the growth endocrine axis that seems to be more related to changes in metabolism, stress resistance, or energy mobilization, than directly to growth (146). However, overexpression of *gh* or *igfs* can indirectly lead to an increment of growth as a side effect. This increment in growth should be taken carefully because, instead of a positive effect, it could mask developmental problems in fish with later repercussions at a population level.

## 6 Concluding remarks

The main factors that influence somatic growth are nutrition, temperature, photoperiod, salinity, and reproduction. Food restriction reduces growth, as well as changes in temperature or photoperiod and reproduction modify feeding, influencing the GH/IGF axis (23, 29, 161, 353). The temperature–size response found in aquatic ectotherms (16) that may involve the animal’s capacity of capturing oxygen and the adjustments in respiratory surfaces have received high attention, leading to an intense debate (19, 20), and several mechanisms have been purposed to explain this fact (18, 20, 354–357). However, these proposals overlook the endocrine regulation of growth and the effects of hypoxia on the GH/IGF axis as well as in feeding and metabolism which will affect somatic growth as well, as described in early sections. Nevertheless, there is an intricate relationship between the abovementioned environmental factors, growth, feeding, and the endocrine processes governing them. These relationships were molded over millions of years through fish evolution and currently are being altered as a consequence of, for example, global climate change phenomena.

The effects of reproduction on somatic growth cannot be explained only as a mere allocation of energy in gonads, instead of somatic growth (20, 161), as many times simplistically illustrated [see (20) and references therein]. The relationship between growth and reproduction would not depend solely on the amount of energy devoted to gonadal development. Instead, it will depend on the hormonal control of each process and the crosstalk between the endocrine axes. Thus, sex steroid hormones may have divergent effects on the GH/IGF axis depending on specie and gender (161, 358–364). Nevertheless, in a general view, they contribute by reducing somatic growth while inducing gonadal development and sexual behavior that may reduce energy partitioning to somatic growth processes. Both GH and IGF have been implicated in gonadal development as well (23, 29, 365, 366); therefore, gender and sexual development produced a discordant variation of somatic growth and GH/IGF axis regulation (161). Sex steroids, particularly estrogens, increase pituitary GH content and release and reduce GH responsiveness and IGF production in the liver [for review, see (29)]. Thus, estrogens uncouple GH levels with somatic growth in a similar fashion to fasting and stress (29). The presence of steroid hormones and its derivates in aquatic environments as a consequence of anthropogenic activity is provoking disruptions in fish that have been mostly studied at the reproductive level. However, taking into consideration all the evidence exposed in this review, it would be important to look at their impact on growth and feeding behavior as well.

Although the available information on the effects of pollutants on growth and feeding is scarce, the few studies that address the issue clearly point out that pollutants would affect growth, metabolism, and development, affecting the animal’s performance. Thus, some heavy metals, EEs, and micro- and nanoplastics have been shown to affect growth and/or the GH/IGF axis. Nevertheless, more studies are required to analyze pollutants’ effects on growth and feeding and their regulatory endocrine network. It is possible, however, to hypothesize that most of the effects of abiotic factors that influence growth and feeding would have a common effector in the HPI axis and in the stress response. The physiological result due to stress and activation of the HPI axis is an increase in serum levels of cortisol. As such, the magnitude and duration of cortisol variation may be used to define the response to a stressful condition (161, 367). There is extensive research on cortisol response to stress, as well as cortisol effects on growth and feeding in fishes (367–371). In a general plot, stress and cortisol reduce somatic growth and appetite and decrease IGF plasma levels or mRNA levels (23, 29).

Future work analyzing the effects of pollutants and other abiotic factors not only on size, condition factors, growth rates, and food intake but also on some key regulatory hormones of somatic growth (GH, IGF), feeding (ghrelin, nesfatin-1), metabolism (insulin), and stress (cortisol, catecholamines) will help to understand the mechanistic control and its perturbation evoked by environmental factors in fishes. All this information would be very useful to integrate the early signals of endocrine disruption with the apical effects provoked by pollutants. This will generate new approaches for the study of the effect of contaminants on fish and the possibility to add a predictive value to the results.

## Author contributions

Both authors, LFC and JIB, contributed to the idea and design of the manuscript, wrote sections of the MS, revised the drafts, and approved the submitted version.
